# Ionic Liquids as Potential and Synergistic Permeation Enhancers for Transdermal Drug Delivery

**DOI:** 10.3390/pharmaceutics11020096

**Published:** 2019-02-22

**Authors:** Zainul Sidat, Thashree Marimuthu, Pradeep Kumar, Lisa C. du Toit, Pierre P. D. Kondiah, Yahya E. Choonara, Viness Pillay

**Affiliations:** Wits Advanced Drug Delivery Platform Research Unit, Department of Pharmacy and Pharmacology, School of Therapeutic Sciences, Faculty of Health Sciences, University of the Witwatersrand, Johannesburg, 7 York Road, Parktown 2193, South Africa; zainul.sidat@students.wits.ac.za (Z.S.); thashree.marimuthu@wits.ac.za (T.M.); pradeep.kumar@wits.ac.za (P.K.); lisa.dutoit1@wits.ac.za (L.C.d.T.); pierre.kondiah@wits.ac.za (P.P.D.K.); yahya.choonara@wits.ac.za (Y.E.C.)

**Keywords:** ionic liquids, transdermal, synergy, permeation enhancer, chemical, physical, Transdermal drug delivery systems

## Abstract

Transdermal drug delivery systems (TDDS) show clear advantages over conventional routes of drug administration. Nonetheless, there are limitations to current TDDS which warrant further research to improve current TDD platforms. Spurred by the synthesis of novel biodegradable ionic liquids (ILs) and favorable cytotoxicity studies, ILs were shown to be a possible solution to overcome these challenges. Their favorable application in overcoming challenges ranging from synthesis, manufacture, and even therapeutic benefits were documented. In this review, said ILs are highlighted and their role in TDDS is reviewed in terms of (a) ILs as permeation enhancers (single agents or combined), (b) ILs in drug modification, and (c) ILs as active pharmaceutical ingredients. Furthermore, future combination of ILs with other chemical permeation enhancers (CPEs) is proposed and discussed.

## 1. Introduction

Currently, ionic liquids (ILs) are a class of compounds under intensive investigation for a multitude of applications including, but not limited to, green chemistry, chemical synthesis [1], catalysis, lubricant fluids [2], plasticizers, organic solvent replacement [3], electrochemistry, and bio- and nano-technologies, among many more [1,2,3,4,5,6]. However, of particular importance is the application of ILs for biomedical applications [4]—more specifically transdermal drug delivery [4]. Based on the reported use of ILs as chemical permeation enhancers (CPEs), there is continued interest for ILs in transdermal drug delivery. ILs were shown to enhance transdermal transcellular and paracellular transport, bypassing the barrier properties of the stratum corneum (SC), employing mechanisms such as disruption of cellular integrity, fluidization, and creation of diffusional pathways and extraction of lipid components in the SC [7,8].

The ability for room-temperature ILs (RTIL) to be modified for various purposes allows for them to be used in many settings, such as solubilizing agents with the ability to solubilize a wide variety of compounds [4], or as permeation enhancers which act on biological membranes leading to improved efficacy and clinical outcomes [9,10,11,12,13,14]. Early work done by Moniruzzaman et al. [15] (2010) showed that IL soluble drug acyclovir can form stable IL–oil microemulsions with dimethylimidazolium dimethylphosphate (MMIM^+^)(MMPO_4_^−^), allowing for an alternative solvent system to be applied when solubilizing drugs with low solubility in organic solvents. Building on this work, Moniruzzaman and co-workers (2016) also applied the solubilizing properties of ILs to biomolecules such as proteins, enzymes, and DNA [16]. Moreover, etodolac, a poorly water-soluble drug, was formulated with 1-butyl-3-methylimidazolium hexafluorophosphate (BMIMPF_6_) to give an ionic-liquid-in-water (IL/w) microemulsion (ME) [17].

The solvating power of ILs is remarkable, leading to their use not only in topical systems, but also oral systems, and their applications in drug delivery formulations are not only limited to solubilizing agents. They were also used to overcome synthesis challenges by inhibiting unwanted polymorphs in crystalline active pharmaceutical ingredients (APIs). Enhanced therapeutic outcomes were noted when strategies such as formulating APIs as ILs (API-IL) or the use of pharmacologically active ILs were applied, including, but not limited to, anti-bacterial, -viral, and -fungal activity, cytotoxic agents, and biofilm-disrupting agents.

This review provides an account of the successful implementation and application of ILs in current transdermal systems and how they can be leveraged for enhanced outcomes, along with their applications in all phases of drug delivery including fabrication of transdermal systems, as an excipient to enhance formulations providing permeation, and even as therapeutic moieties to improve disease outcomes. This review is based on mechanistic, computational, and structure–activity evidence for their use in improving permeation and strategies for synergistic combinational therapy to that end.

### A Brief Note on Deep Eutectic Solvents (DES)

Both DESs and ILs are touted to be greener solvents than current industrial standards; they are similar in nature with the key difference that can be found in the components used. In DESs, an organic halide is complexed with an organic agent, most often a hydrogen-bond donor derived from non-ionic species [18,19]. Similarities between both are significant, including their applications as modifiable solvents where DESs are gaining more and more traction over ILs due to a host of factors. The most notable reasons include decreased cost, relatively easy preparation, lack of water reactivity (a major disadvantage of some of the earlier ILs), non-toxicity, and biodegradation pathways (a major advantage as several ILs show environmental damage) [18,19,20]. The hydrogen bonding seemingly confers many advantages similar to ILs, providing compounds that have low vapor pressure, dipolar nature, low volatility, and more [18]. While they are currently being applied to transdermal delivery systems, DESs are beyond the scope of this review and are, therefore, omitted.

## 2. Properties of ILs

ILs are an interesting group of chemical compounds composed of ions which have melting points below 100 °C [21,22,23]. ILs are generally described as having ideal properties, such as low to negligible vapor pressure at room temperature, extensive and varying solubility profiles, high thermal stability, non-flammability, adaptable polarity, inert chemical profiles, exceptionally low melting points (for ionic-bonded compounds), variable viscosities, and many more customizable properties [14,21,23,24]. Pioneering work by Paul Walden in 1914 established ethyl-ammonium nitrate as a prototype IL which was later succeeded by a multitude of alternative generations of ILs over several decades [1] with the emergence of new tailored and greener ILs [21].

While ILs are often discussed as a group and are attributed relative group properties, it is essential to note that the sheer diversity of ILs [23] means that there are very few descriptors that fit all ILs [22]. They can be modified to provide a variety of possible properties that are desired. They are made up of two distinct components—the anion and cation moieties. The cation is generally bulky and organic in nature, whereas the anion is relatively much smaller and inorganic. This combination leads to a decrease in the crystallinity of the system, allowing ILs to be liquid at such low temperatures and confer any number of favorable properties as mentioned above.

**Figure 1:**
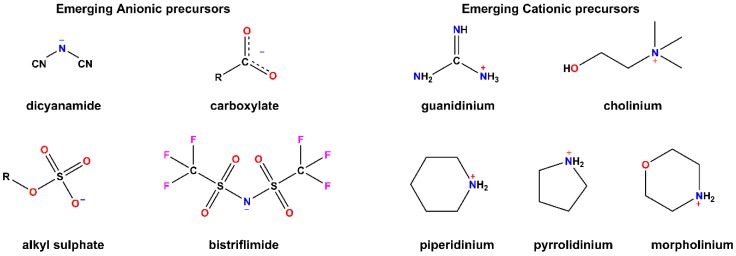
Emerging anions and cations used in ionic liquids (ILs).

The make-up of ILs means that any number of permutations are possible so long as a cationic and anionic species with poor crystalline packing exist. This led to the discovery of many nascent species. Included among these newer IL bases (Figure 1) are cholinium and guanidinium cations, which confer the advantage of being biodegradable and are, therefore, referred to as bioinspired ILs (BILs) [25]. The more traditional base cations include quaternary ammonium, imidazolium, pyrrolidinium, pyridinium, and phosphonium cations. Some of the newer species, such as metal-containing cations, no longer fit in the traditional scope of ILs where organic cations and inorganic anions are combined. These new metal ILs are formulated using metal-containing cations and various anions, allowing the formation of IL-like salts. A further subset of these metal-containing ILs involves those with magnetic properties [26]. The abovementioned cation species and new alternatives increase the scope for application of these ILs in biomedical research—particularly bioinspired IL bases.

### 2.1. Chemical Traits

Due to the sheer number of possible permutations of these constituents that make up ILs, it becomes very difficult to investigate possible chemical effects [14]. However multiple resources including, but not limited to, computational models, databases such as IL-THERMO, and studies of effects (using theoretical methods. i.e., classical and density functional theory (DFT) molecular dynamics modeling), gave insights into chemical properties. 

Recent studies involving the structural properties of ILs showed that ILs contain micro- and nano-domain organizational structures [27], and ions tend to self-assemble into subdomains forming amphiphilic nano-structures which persist. Based on these structural features, ILs seem to resemble more of a nano-heterogenous substance which is a largely unique characteristic of ILs and is not found commonly among solvents and other dissolution media. Spatial studies on these domains are extremely difficult and are modeled theoretically using classical and DFT molecular dynamics. The work is confirmed by spectral studies and accounts for difficulties when certain ILs are characterized by nuclear magnetic resonance spectroscopy (NMR), thus requiring external forces throughout the characterization process disrupting ultra-structures [27].

Hydrogen bonding and ionic interactions are the key drivers in structural organization found in ILs. A core difference between ions of inorganic salts and ILs is the size of the participant ions. Inorganic salts consist of spherical species allowing neat and ordered packing, in contrast to the large and bulky cation of ILs with the charge being distributed over a much larger area. This in turn influences the local structure of the IL, and minute changes can lead to drastic changes in the activity demonstrated readily by cation choice. As an essential component, the cation influences factors such as viscosity, conductance, electrostatic forces, surfactant properties, polarity, aggregation challenges, and toxicity profiles, among others. A simple variation such as chain length can have extreme influences on the physio-chemical properties of the resulting ILs (as outlined in Table 1).

The ultra-level organization of ILs is only possible due to the large bulky cation. The charge distribution over a much larger area allows for lower charge density and thwarts electrostatic repulsion. This convergence creates noncovalent interactions, leading to the structural properties observed in ILs.

The application of ILs as solvents is a promising trend in current research, and the solubility of ILs is a key concern. Solvent–solute interactions for ILs are often specific rather than explained in non-specific class terms. The ability for ILs to be miscible with a wide variety of other solvents provides an ideal solution for dissolving most solutes. The general mechanism for IL solvation is not yet clearly understood, as some undergo H-bonding, others show dipole–dipole interactions, and some even undergo π–π interactions. An interesting find by Visser et al. [28] (2002) showed that neutral substances dissolve with ease as opposed to ionic species; however, with optimization, they are capable of dissolving most substances to give ideal miscibility in reasonable molar ratios [29].

### 2.2. Physical Properties

Physical properties of ILs are as diverse as chemical properties, and most of these characteristics can be configured and fine-tuned for a given application [12]. Among the most important properties is the density of ILs. The density of ILs ranges between 0.9 and 1.7 g·cm^−3^ which renders ILs to be relatively more dense than conventional organic solvents [34]. Density can be affected by temperature, pressure, and, most importantly, molecular mass and the interactions between molecules. As such, it was found that larger organic cation-containing ILs have a lower density [29]. 

Another important property affecting the application of ILs in TDD is viscosity. The viscosity of a given IL is a very important property, which varies from <10 to >1000 cP at room temperature. This characteristic of ILs limits the possible applications of ILs where they are used as solvents. Viscosity for ILs is expressed as a viscosity co-efficient rather than a stated value; this in large part is due to ILs not following Arrhenius behavior. The alternative, Vogel–Tammann–Fulcher (VTF) equation, accounts for an additional factor, i.e., the glass transition. 

The lower melting points of ILs despite their inherent ionic nature are among the more fascinating properties of ILs. Due to this property and low vapor pressures, they are extremely attractive alternatives to current organic solvents. RTILs are defined by melting points at or below room temperatures, and variances are accounted for due to charge distribution, H-bonding capability, and symmetry. Freezing-point calculations are often experimental and unreliable as ILs undergo variable rates of supercooling. Factors affecting these properties were experimentally found by Katritzky et al. [35] (2002), and include molecular shape, symmetry, rotational freedom, and electrostatic interactions. 

Polarity features a key role in solvent–solvate interactions. As explained for each of the properties above and for ILs as a whole, there are no unifying rules; rather, there are structure-related changes that can be tuned and optimized for a given task. Solvatochromic dyes gained favor in polarity studies where a variety of compounds such as Nile-red and betaine-30 have different absorption and emission bands based on the solvents in which they are dissolved. Key features affecting the polarity of ILs include chain length variation, whereby long and branching chains are more hydrophobic. 

### 2.3. Interactions of ILs with Biomolecules and Membranes 

The organic character and increased use of ILs in industrial processes inspired the first studies of ILs on bio-molecules and bio-organisms. While several studies highlighted toxicity and their negative impacts, these can be applied in a variety of ways to be advantageous. The possibilities include, but are not limited to, anti-bacterial properties, and the stabilization and storage of bio-molecules such as proteins, enzymes, and DNA. More impressively, the abilities of ILs to re-functionalize defective amyloid fibers, dissolve complex polysaccharides, and create pores in bio-membranes, among many others were reported [4,32,36,37,38,39,40]. These processes can yield merits such as selective bacterial killing, selective toxicity to cancerous cells, and application for transdermal systems, respectively. 

**Figure 2:**
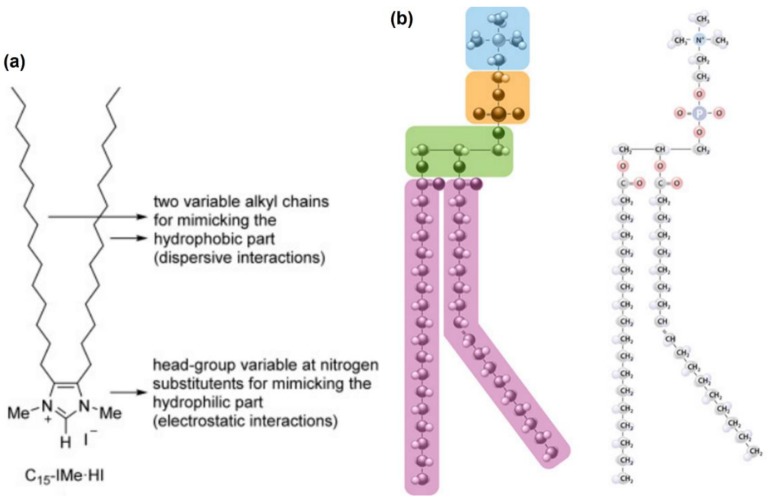
Sketches depicting (**a**) double-tail lipid-mimic imidazolium-based IL; (**b**) glycerol–phospholipid sub-regions with large tail lengths. The similarities are striking and allow for their ability to intercalate within the phospholipid membrane structure. Adapted with permissions from (**a**) Reference [41] John Wiley and Sons, ©2015, and (**b**) https://www.nature.com/scitable/topicpage/cell-membranes-14052567 Nature Education ©2010.

Any initial reaction will occur at the interface between a bio-membrane and the ILs. It is critical that the interaction must be safe for human use. These membranes serve not only as a protective barrier, but are also key in regulating diffusional processes and cell replication. The most accepted model of bio-membranes is the phospholipid bilayer, and testing employing ILs investigating their effects employs this model. Several IL bases show significant resemblance to phospholipids and can be synthesized to resemble biologically based materials, as demonstrated by Wang et al. [41] using dimethylimidazolium iodide derivatives (Figure 2). In this study, the similarities extend to include an ionic polar character and internal hydrophobic regions. Increasingly, ILs are being based on biological materials such as those reported in the previous study and other work done by the same authors [42]. Bio-inspired ILs are different to biomimicry ILs such as those reported by these previous studies. Bioinspired ILs such as those reported by Benedetto and workers (2014) [43], where choline-based cations and several amino-acid–anion pairs were modeled and evaluated for biological systems as starting materials. This led to significant similarities between endogenous groups and these artificial substrates that can be observed. 

**Figure 3:**
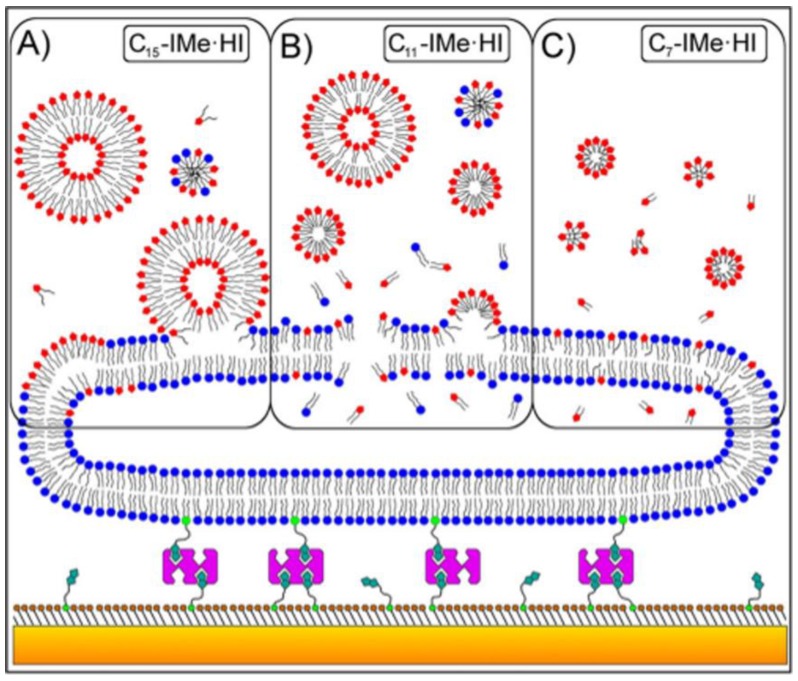
A model of the interaction between ILs (imidazolium-based) and bio-membranes where a gold-coated sensor surface (orange) has a chemisorbed self-assembled monolayer (brown) tethered by biotin linkers (green) to streptavidin which also tethers liposomes (blue). Panel (**A**) depicts a vesicle, which can then associate and intercalate into the bilayer membrane. Panel (**B**) focuses largely on mechanisms causing lyses to the membrane by forming vesicles or micelles in an aqueous medium, thereafter disrupting the membrane structure. Panel (**C**) focuses on mechanisms that allow pass-through within the membrane by disassociating into single molecules. Adapted with permission from Drucker 2017 [48]; © 2017, American Chemical Society.

Simple biomimicry between IL structure and endogenous phospholipids, however well done, does not assure safety and compatibility of ILs in biomedical applications. This is evident by the work of Evans et al., where it was reported that slight changes in the cation chain when using 1-butyl-3-methylimidazolium chloride led to substantial damage to the bio-membranes that were investigated [44,45]. These experiments were limited in their scope, and work done by Benedetto et al. [46] further explored the penetration of 1-butyl-3-methyl-imidazolium chloride (BMIMCl) and choline chloride (Chol^+^)(Cl^−^) ILs in bio-membranes by means of neutron scattering. This work demonstrated that phospholipid bilayers retain their two-dimensional (2D) characteristic configurations at concentrations up to 0.5 M. This work gave significant insight into changes to the bio-membranes which included the shrinking thickness of the bilayer, the accumulation of IL cations within the junction between the polar heads and hydrocarbon tails, and the lipid bilayer composition affecting the amount of accumulation that occurs, as well as the degree of penetration, but not the location of accumulation. Further studies such as crucial work done by Jing et al. [45] also confirmed this bioaccumulation of ILs causing swelling and also showed that concentration was a key concern and could completely disrupt the bilayer. Building on their previous work, Benedetto et al. [47] included molecular dynamics simulations which verified the validity of the results, and a potential mechanism of interaction of ILS with bio-membranes (Figure 3) was proposed.

Effects of ILs on bio-membranes are affected by several factors. The concentration of ILs applied affects the bio-membranes and results in shrinkage of the bilayer and increased the elasticity of the bilayer. Disruption of phospholipid membranes is affected by the hydrocarbon chain length in the cation. The interaction of cholesterol-containing bio-membranes makes them more resistant to rupture, but at a threshold concentration; once exceeded, the effects on these cholesterol-containing bio-membranes are much more apparent. Concentration and chain length play critical roles in cytotoxicity, as they allow easier penetration into the membrane. Short-tailed ILs spontaneously insert into the membrane, but long-tailed ILs self-assembled into micelles that are eventually absorbed and form a monolayer in the upper portion of the bilayer. This means that short-tailed ILs are more mobile and can diffuse more easily than their long-tailed counterparts.

## 3. Current Challenges in Transdermal Drug Delivery

Transdermal drug delivery is yet to be fully appreciated, as more studies are required to fully realize its full potential [49]. The major challenge associated with transdermal drug delivery is the almost impermeable barrier created by the SC measuring 10 μm in thickness with variances in different parts of the body [50,51,52,53]. While steady work is being carried out in this field, there are still very few APIs that are likely candidates for delivery through this route. Permeation of APIs is historically the biggest challenge in transdermal drug delivery, with multiple generations of transdermal delivery systems [49] trying to overcome this without much progress to show [54,55].

**Figure 4:**
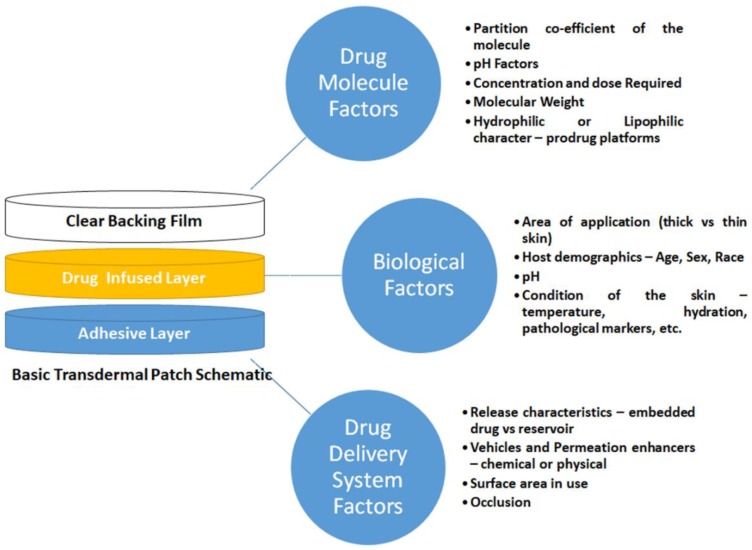
Depiction of the most critical factors to consider when designing a transdermal drug delivery system.

Transdermal or percutaneous absorption refers to the rate of absorption of a topically applied chemical. The necessity for determination of this rate is twofold the effectiveness of transdermal application, thereby avoiding many of the disadvantages associated with other modes of drug delivery such as oral or parenteral routes. With so many factors (Figure 4) affecting transdermal drug delivery such as the method and location of drug delivery, the drug molecule itself, and factors such as inter-individual variances (skin age, condition, and blood flow), it becomes apparent that simple zero- or first-order kinetics are not sufficient to describe it. A key consideration in effective drug delivery systems is protecting the drug moiety from undesirable metabolism while enhancing transport to active sites. Biological and physiological hurdles prevent efficient drug delivery, particularly the skin in transdermal systems. 

Skin is the primary barrier to any transdermal drug delivery. Designed to be impermeable and extremely adapted to its function [56], the outermost layer (the SC) is the most impermeable layer and extremely well differentiated, derived from keratinocytes that are terminally differentiated. A lipophilic matrix that anchors corneocytes made up of free fatty acids, cholesterol, and ceramides provides the only diffusion phase to allow drug delivery to occur into the subcutaneous layers [57,58]. Freeze-fracture electron microscopy showed that the SC is arranged in lamellae, which is facilitated by the presence of cholesterol sulfate. The matrix is extremely heterogeneous, made up of 11 classes and 342 individual ceramide species alone which were identified by Masukawa and co-workers (2008) [59].

Flux across the skin for molecules that are natively permeable can be simply expressed as

(1) $$J=K_{p}\cdot \Delta C,$$

where *J* is the flux (expressed usually as µg∙cm^−2^∙h^−1^), *K* is a co-efficient denoting the permeability of the skin with regard to the permeant in question, and ΔC the concentration gradient across the barrier. Where passive diffusion is the primary means of permeation, $K_{p}$ is defined by the partition co-efficient (*P*) and the diffusion co-efficient (*D*) and the thickness of the boundary (*h*), mathematically expressed as

(2) $$K_{p}=\frac{P\cdot D}{h}.$$

To date, many techniques and methods were evaluated for improving permeation, and none of them can be labeled as ideal. The criteria [60,61] to be labeled as ideal is quite significant and include a varying number of properties. These properties range from chemical nature, such as non-toxic, non-irritant, and non-allergenic, to ideal conditions such as rapid activity and inert physiological profile. This list also includes mechanistic criteria, such as unidirectional biased permeation, i.e., allowing drug molecules in but not letting that which is in the body out, and rapid recovery of skin when the agent is removed. Formulation application is also considered, which must meet standards such as compatibility with a variety of formulations and being cosmetically acceptable for patient compliance.

The criteria for drug molecules suitable for transdermal drug delivery are extremely stringent and include, but are not limited to, an aqueous solubility greater than 1 mg/mL, an oil–water partition co-efficient between 10 and 1000 (at the same time, it cannot be too lipophilic as then it will remain for an extended period in the subcutaneous layer), molecular weight under 500 Da allowing it to be sufficiently mobile, and a melting point under 200 °C with a dose not exceeding 10 mg, i.e., potent drug molecules [57,62,63]. These are preliminary ideal conditions for passive drug delivery across the skin barrier. One of the most widely used indicators is the partition co-efficient described by Potts and Guy [64] denoting hydrophilicity, with optimal results being seen at values ranging from (1)–(3).

(3) $$\log K_{p}=0.71\cdot \log P_{Octanol-water}-0.0061\cdot Molecular\;Mass-2.74.$$

With only a few drug molecules such as scopolamine, nitroglycerin, clonidine, estradiol, fentanyl, nicotine, testosterone, and norethisterone being able to meet the stringent criteria necessary for TDD, it becomes very apparent that the limitations to this system are vast but not without their own advantages. The skin provides a vast surface area for absorption of ~1–2 m^2^, decreased metabolism of drugs, enhanced patient compliance due to several factors (non-invasive, reduced side-effect profiles [65]), painless, and extended drug release systems especially for short half-life drug molecules decreasing patient interventions, among others [14,57,63,66]. Those advantages are patient-driven and quite substantial; however, these systems also decrease the need for skilled healthcare practitioners especially in rural settings, and produce limited hazardous waste materials [67].

Three major techniques (with a few pertinent examples listed in Table 2) are used to improve permeation across the skin. The first is formulation enhancement, whereby a variety of formulation modifications and strategies are used to help the delivery system achieve permeation. These strategies are not always applicable and can only be used for those drug molecules which are natively permeable through the skin. The second is physical permeation enhancement. This usually involves usually devices of some sort that disrupt the skin barrier by physical means by producing pores which drug molecules can permeate through. These systems have the disadvantage of needing a device which the patient must carry around; alternatively, the device may cause punctures or cavitation, which carries a risk of infection [68].

The last and most investigated methods are CPEs. This method involves the use of chemical compounds that interact with the skin and affect the activity of the barrier. Various functional groups in these chemicals interact with the skin and its lipid content causing disfiguration to the skin layer. Chemical enhancers of this nature are liable to cause skin irritation, as permeation activity is proportional to irritant activity.

### Current Limitations of Chemical Permeation Enhancers (CPEs)

The most extensively used method of permeation enhancement involves the use of chemical moieties that would interfere with functional groups in the skin in a reversible manner, thereby compromising barrier activity in the SC. This method can be applied to permeate otherwise impermeable drug molecules and to significantly enhance those that already can permeate through. When considering chemical permeation enhancement, several factors play a key role. The factors can be narrowed to the steady-state flux $\left(\frac{dm}{dt}\right)$ measured by the mass (*m*) passing per unit area of membrane in time (*t*), concentration of permeant (*C*_0_), partition co-efficient (*K*) between the membrane and application, diffusion co-efficient (*D*), and membrane thickness (*h*), expressed as [78]

(4) $$\frac{dm}{dt}=\frac{D\cdot C_{0}\cdot K}{h}.$$

The advantages of using CPEs over other types of enhancers include factors such as design flexibility, self-administration, patient compliance, and easy incorporation into formulations [79]. The inherent limitations to CPEs are quite significant, especially when considering that high-molecular-weight compounds are precluded. Many recent advances are based on peptides and protein deliveries which are, by their nature, large in size and often charged species. This list does not preclude the use of multiple agents (including physical permeation enhancers) to act synergistically [54]. Many are used in combination to potentiate the effects of each other such as the combinations of ethanol and propylene–glycol. However, each of these chemicals has the potential to be irritant to the skin [63]; therefore, the use of some of these agents and any combination increases the risk of skin irritation that may be deemed clinically unacceptable. Chemical enhancers that are found in marketed dermatological products are usually alcohols (ethanol), propylene glycol, and sodium lauryl sulfate [57]. 

## 4. ILs Meeting the Needs in Transdermal Drug Delivery

### 4.1. ILs as Skin Permeation Enhancers

**Figure 5:**
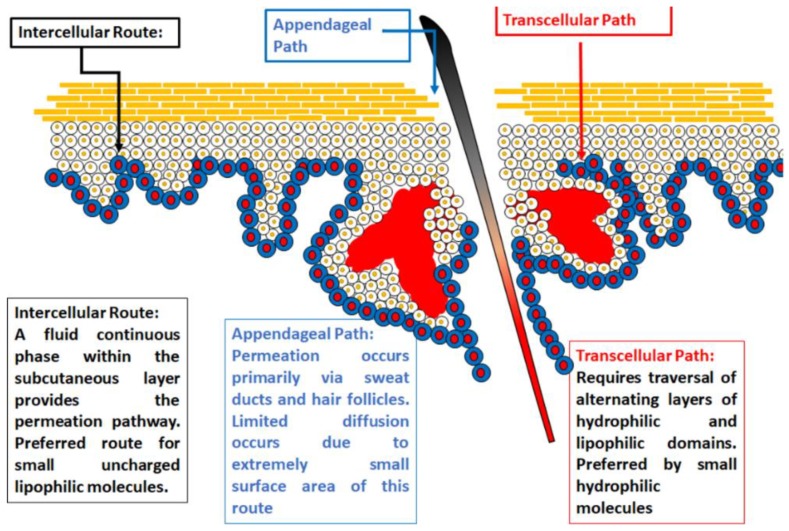
Depiction of the possible routes of transdermal permeation which lead to effective transdermal absorption.

ILs were shown to enhance permeation of drugs through the skin [15,40,80,81,82,83]; therefore, many research studies were conducted to account for their underlying mechanisms of action. Several mechanisms that are largely dependent on the chemical make-up of the IL were proposed. Most notably, the work done by Monti and colleagues showed structure to have a large impact on the degree of permeation [84]. A key factor related to permeation enhancement is the physicochemical properties of ILs. Recent research suggests electronic profiles of the ILs have a large part to play in their permeation activity [85]. However, this mechanism is extremely broad and does not account for all IL permeation enhancement profiles. Broadly classified, all ILs with permeation enhancement are hydrophilic or hydrophobic. Hydrophilic ILs act by opening tight junctions within the SC, thereby promoting paracellular transport (Figure 5) acting by enhancing fluidization mainly within protein and lipid regions, whereas hydrophobic ILs improve partitioning into the epithelial membrane by providing channels, thus promoting transcellular transport in the lipid regions [25]. Among the best documented is the activity of 1-octyl-3-methylimidazolium-based ILs which act by disrupting structural integrity by inserting into the membrane [7]. It was also demonstrated that ILs possess the ability to fluidize cell membranes, particularly seen with hydrophilic imidazolium-based ILs [8,40], as well as lipid extraction in the SC. Transdermal delivery of several unlikely drug candidates such as protein molecules [86,87], methotrexate [88], and acyclovir [89], among others benefited greatly from IL incorporation, opening the way for a multitude of alternative possible drug molecules. 

Toxicity is a crucial factor to consider when looking at the medical or biomedical applications of any technology. Older generations of ILs were not suitable for medical applications. To overcome this drawback, bioinspired ILs were designed and were shown to give desirable biodegradation and decreased toxicity profiles. Choline-based bioinspired ILs are currently the most intensively investigated [40,90]. Cytotoxicity studies conducted, usually focusing on ecological toxicity, do indeed provide a reason to be concerned, especially amidst claims that some ILs are more toxic than current standards [91]. Toxicity correlation exists, whereby longer chain lengths are found to be more toxic, as well as effects due to the presence of pharmacological activity, the present ionic species (anion and cations), and the presence of oxygen in the compound. Limited studies were performed in an effort to gauge toxicity on humans, and they include enzymatic assays (acetylcholinesterase (AchE) and AMP deaminase), as well as cytotoxicity studies against cancerous cell lines (colonic, cervical, and breast) [92] and some primary human cells such as normal human bronchial epithelial lines [40]. 

However, these abovementioned studies show toxicity on single-cell organisms, which can be exploited for antimicrobial properties [40]. Additionally, the use of ILs as adjunctive treatments or alternatives to chemotherapy on cancer cell lines is possible and was reported in Reference [4]. Cholinium-based ILs were proven to be much less toxic than regular ILs, whereby effects on AchE are much more limited, and persistence in the environment is limited by the rapid and substantial biodegradation [14,93]. These cholinium bioinspired ILs have limited or no applications in antimicrobials or antineoplastic agents. 

The mechanism through which ILs can be beneficial to transdermal systems is not only limited to their permeation enhancement. They can play roles such as surfactants and optimize the thermodynamic activity of drug molecules, they can act as efficient solubilizing agents or cause fluidization of the lipid bilayer, and they can even disrupt the matrix by acting on keratin fibrils. The permeation activity is high with limited cytotoxicity, which fulfils two of the criteria for ideal permeation enhancers [84].

### 4.2. Evidence of Successful Synergy between Combinations of ILs and Chemical Enhancers

Synergistic combinations of ILs with other chemical enhancers are limited and mostly experimental in nature. The majority of IL applications do not lie in medical and biomedical use; thus, sparse work was done beyond proving IL effectiveness as a penetration enhancer, and limited toxicity studies make the investigation of ILs in medical applications seem preliminary [94]. Most combinational studies look at ILs, such as use as solvents, reaction media, and excipient substitution in final formulations. 

The versatility of ILs does not come from their properties, but rather their heterogeneous nature, allowing them to be synthesized from any number of sources. So long as the result is an IL, it will retain many of the advantages of ILs. They can be synthesized from dual APIs, from biologically based ions, and even from normal synthetic compounds and solvents. This means that the proven safety of GRAS (generally recognized as safe) chemicals (such as terpenes and surfactants) can be leveraged as a basis for a safe and effective ILs. This is not to say that ILs are not safe; in fact, one of the major selling points of ILs is safety; however, limited safety data make them risky investments in the current market for commercial applications. 

The current market CPEs can benefit from a variety of ways when incorporated with ILs (as explored briefly in Table 3. The general properties can be improved, as well as enhancing permeation, reduction of unwanted side-effects, and better safety profiles. Successful synergistic combinations can be proposed ad nauseum due to the versatile nature of ILs; however, in this paper we highlight some of the more practical possibilities which are based on previous or extrapolated evidence for their inclusion in pharmaceutical products for higher standards.

IL incorporation with alcohol-based permeation enhancers can lead to the incorporation of a wider variety of APIs via co-solvency, which aids in drug solubility. Similarly, with other permeation enhancers such as Azone or terpenes, limitations when incorporating APIs can be overcome with IL combination. Fatty-acid permeation is potent after a pre-treatment cycle; by using this method and via the incorporation of APIs in IL vehicles, not only can the permeation be enhanced, but the rate of transport can also be increased. Dimethyl sulfoxide (DMSO) incorporation into ILs may overcome limitations such as lowered concentration of DMSO for permeation activity and decreased side-effect profiles; however, the tangible presence of DMSO reduces the discreet profile of the IL and, thus, should be considered in sealed systems. Surfactants are versatile and relatively safe and are, therefore, reused in many formulations. They also boast extremely high permeation enhancement effects; however, the most active permeation surfactants are also extremely irritant to the skin as demonstrated with cationic and anionic surfactants. In this case, finding a balance between irritant properties and activity is achieved with non-ionic surfactants. The incorporation of safer non-ionic surfactants into ILs enhances permeant activity while maintaining safety profiles. Some of the earliest work with medical applications of ILs done by Moniruzzaman et al. [15] showed the benefits of this particular combination. Added to this is the possibility for completely replacing surfactants with ILs, which can act as the surfactant within the system. Vesicle and IL combination is also quite versatile as discussed for surfactants. ILs can be assembled into vesicles with great ease and even act as actives that assemble into vesicles [95,96]. When employing this approach, however, care must be taken when choosing the IL, as the choice of IL will affect bioactivity greatly.

The application of ILs in these systems will greatly enhance their activity, safety, and overall therapeutic outcomes. The above listed incorporations are all feasible with current technologies and are limited only by the diversity of ILs, making rational selection of appropriate combinations extremely difficult. While applications of ILs in other industrial sectors are growing rapidly, the stagnant medical applications are not simply due to research; scarce safety data also decrease the likelihood that formulations with ILs will make it to the open market. This issue can be overcome by conducting more in-depth cytotoxicity cell studies, and in vivo and ex vivo studies, which could lead to the translation of ILs from research applications to the development of drug delivery systems.

### 4.3. ILs in Drug Dissolution for Improving Bioavailability in TDDS

#### 4.3.1. Drug Moiety and Delivery System Modification

As mentioned earlier, the use of ILs as reaction media and solvents is well studied. They can be modified to achieve any number of favorable properties; however, this is not the limit as they can be further used in API synthesis to aid as catalysts and transformative media [101]. Particularly when using biotechnological processes, ILs can act as selective enzyme enablers to improve yields [4] and limit waste produced. The nano ultra-structures (as discussed earlier) provide an ideal environment for selective reactions to favorably alter APIs. While their use as catalysts and solvents helps reduce waste produced, they found a niche when applied to enzymes. They can help with stability, enhance the activity of enzymes, control folding in proteins, and reduce aggregation of the proteins [14]. Most ILs contain some degree of hydration and can further adsorb water and can affect the stability of these macromolecules. 

ILs were applied to modify current emerging delivery systems for effective delivery of APIs. These include ILs acting as functionalizing agents, solvents, dispersing agents, nanoparticles, nanocarriers, and substrate activation. Emulsion modification became a key strategy, as evidenced in work by Kandasamy et al. [89], where ammonium acetate-based ILs promote the ease of formulation of IL-in-oil microemulsions, further improving the stability of the system and enhancing solubilization. This was also evidenced in work done by Yoshiura et al. [88], demonstrating microemulsion size and size distributions are improved by IL incorporation, leading to enhanced transdermal permeation. ILs in these microemulsion-based systems act as solvents, whereby they can solubilize the API. Further to this, they can replace both the hydrophobic and hydrophilic moieties in microemulsion-based systems. The advantages of IL applications in these systems include the solvating power of ILs, which require less solvent and can solubilize a wide variety of hydrophilic and hydrophobic drugs; their use as permeation enhancers is a key feature in transdermal systems [80].

#### 4.3.2. Forming a Complex of IL with the API

ILs can be implemented in a variety of ways to aid in formulation studies. A common strategy to improve absorption and bioavailability is that APIs can be modified and formulated as prodrugs. Prodrugs are inactive biologically until they are bio-transformed into the active metabolite. Advantages of prodrug platforms include increased bioavailability, lowered metabolism, improved site specificity, and controlled release of the active metabolite. ILs were combined with APIs to make prodrug platforms [83] via the addition of hydrolysable ionic groups to neutrally charged APIs. These were then combined with the correct counterions to formulate a new prodrug IL. The advantages to this strategy are (a) the use of a counterion that is also an API leading to a dual-function IL, and (b) combination with a penetration enhancer in transdermal systems to improve permeation [102,103]. 

The prodrug formulation can further be enhanced by combining IL-APIs with these prodrug platforms, thereby conferring the advantages of both strategies. Cojocaru et al. successfully created prodrug API-ILs by adding hydrolysable functional groups to neutral APIs that can be paired with suitable anion counterparts [104]. The authors of the paper did acknowledge that the chosen IL forms used may not be the most suitable and encouraged substitution with those that are more suitable such as carboxylic acids and amines. By using this as a starting point, the advantages can be numerous. Advantages of prodrugs and ILs aside, the pairing of synergistic drugs with appropriate anion and cation pairs (dual APIs discussed later) can lead to the need for less individual medicaments, thereby further enhancing patient compliance. 

Release studies conducted by Cojocaru et al. [104] found that newly developed acetaminophen-based prodrugs had slower release times (at 210 min ~89% release) when compared to the neutral forms (at 210 min >97% release). This slower release profile is not in itself a disadvantage, particularly when considering extended-release systems are becoming more common.

#### 4.3.3. Dissolving the API in the IL (Solvation)

Solvation of an API forms the first stages of drug absorption and is a critical factor to consider in any efficient drug delivery system. Currently, the use of organic solvents in the pharmaceutical industry is an undeniable challenge to sustainable growth in this sector. Further to this is the organic contamination of pharmaceutical products from these solvents. Alternative solvents and strategies are long overdue, in an effort to produce greener and more sustainable practices with lowered waste generation (corresponding e-factors). As such, ILs provide a suitable alternative to volatile organic solvents for a variety of applications, including use as a reaction medium. The advantages they offer are numerous and include improved solubilization [105], faster reaction rates, higher purity end products, decreased waste generation, and less fastidious reaction conditions. The preferred cation in these reaction media is methylimidazole combined with anions such as BF_4_, PF_6_, and NTf_2_. This combination and other ILs can be used in the preparation of a variety of drugs, including antiviral agents, non-steroidal anti-inflammatory drugs (NSAIDs), anti-neoplastic agents, and anti-infectives.

For an API-solvent combination to be used in medicines and bio-medical applications, its solubility must be greater than 1 mg/mL. Many IL candidates may exist meeting this standard for any given API, but their toxicity profiles remain to be investigated [14]. This is a significant impediment to the widespread use of ILs in medical and biomedical fields. Limited toxicity studies were conducted, and much more work needs to be done in this field, as can be seen by in-depth meta-analysis studies such as those conducted by Heckenbach and co-workers [106], where many studies dealing with ILs (~39,000) were reported on, but a significantly lower number (~220) deal with toxicity in ILs. By using biologically derived bases, the compatibility can be improved, and the toxicity can be limited [94]. Bioinspired ILs have limited issues with toxicity as they can be metabolized and excreted; further modifications such as alkyl group substitution with ester groups further reduce toxicity. IL blends were also used to accommodate and alter varying solubility standards. Using water-miscible ILs in combination with hydrophobic ILs allows a degree of freedom when considering hydrophobic APIs. 

### 4.4. ILs as Active Pharmaceutical Ingredients (APIs) in TDDS

APIs in large part exist as crystalline solids due to historical reasons, better stability, and support from guidelines such as the Food and Drug Administration (FDA) guidelines, which give all the necessary information for solid crystalline API analytical procedures, and decision trees providing streamlined manufacturing processes. This is not the same for other API forms. The disadvantages associated with these crystalline solid APIs affect many physicochemical properties and, ultimately, the behavior of these compounds when introduced in medicaments. The most apparent and problematic challenge is polymorphism [14]. Polymorphs occur when crystalline substances crystalize in more than a single form. This can lead to challenges in solubility and absorption, ultimately affecting bioavailability. Polymorph forms can be affected by manufacturing conditions such as solvent choice, temperature, pressure, and mechanical stress. Polymorphs do not only occur during manufacture, but also during long term storage, where less stable polymorphs will revert to more stable polymorphic structures under storage conditions. While stable polymorphs are less prone to degradation, they are often more difficult to solubilize and, when dissolved, they can salt out.

Challenges such as polymorphism, solubility, and bioavailability can be overcome by using ILs [14]. Historically, the first API-ILs were considered to be miconazole derivatives [4]. Building on this work, the formation of ranitidine docusate, lidocaine docusate, and dual API-ILs was later reported. These studies led to (a) the synthesis of didecyldimethylammonium bromide and sodium ibuprofen to give didecyldimethylammoniumibuprofenate, and (b) a demonstration that the API could be either the anion [107], cation [108], or both [82]. Work done to date (pertinent examples illustrated in Table 4 include the use of lidocaine, sulfacetamide, ibuprofen, cinnamic acid, piperacillin, acetyl-salicylic acid, and penicillin G [14]. The use of IL-APIs not only helps overcome manufacturing challenges, but also positively impacts therapeutic outcomes by improving API bioavailability and penetration, as well as providing alternative delivery methods and beneficial synergistic interactions.

## 5. Proposed/Future Opportunities for Synergism in TDDS

The incorporation of ILs in the abovementioned systems can be summarized to a few key strategies for future deployment, as follows:

1) The use of ILs in a pre-treatment cycle: By pre-treating with ILs, the skin barrier properties are decreased. This in turn would allow more of the active compounds being employed with physical permeation to penetrate.
2) Using ILs in the permeation system: ILs are a diverse medium in which permeation systems can be made and modified. They allow incorporation into systems by acting as electrolyte solutions, coating media, and media in which delivery systems can be synthesized. Care must be taken to ensure that unwanted products do not form when changing the manufacturing process.
3) The use of ILs as a bioactive: Certain drug molecules can be derivatized as ILs and can be incorporated into other permeation systems. This will improve permeation and remove unwanted polymorphs. Using prodrug platforms in this method may enhance permeation; however, the need for the molecule to be activated may lead to longer therapeutic lead times.
4) Synergistic combination: A primary method of improving permeation with chemical enhancers is the combination use of ILs with another chemical enhancer, which can lead to synergistic or additive penetration. They can be combined in a single formulation where the IL can be the primary solvent, co-solvent, or surfactant, or can be employed as a second permeation enhancer. The risk to this strategy is that the irritation may be much more apparent during patient use.
5) Altered favorable environment for drug molecules: The use of ILs as a medium for solubilizing drug molecules can be varied to be more favorable. This strategy may not be applicable to a large number of drug molecules, as most are synthesized as crystalline solids as opposed to liquids. These classes of drug molecules can therefore be easily incorporated into the ILs.
6) Diverse activity profile: ILs can act as a number of formulation components. They can act as vehicles solubilizing drug molecules, surfactants, micelles, and permeation enhancers, and they can replace aqueous or oily phases in formulations. This diverse portfolio makes them ideal for incorporation into many formulations. However, a lack of long-term toxicity studies limits the widespread use of ILs.
7) Modification of embedded substrates: The use of ILs to enhance drug profiles has sound evidence. These are often favorable for drug delivery where drug molecular profiles are altered to enhance permeation and systemic absorption. The long-term stability and safety of these altered drug molecules is largely unknown and, therefore, requires much work before this strategy can reach clinical applications.

## 6. Conclusions

The benefits of transdermal drug delivery are apparent, and a significant number of potential techniques already exist. However, systemic absorption of most drug molecules is still elusive due to the skin and its barrier properties. The possibility of using ILs as transdermal delivery systems or permeation enhancers, and even in synergistic combinations without the risk of major toxicity is a trademark of their versatile nature. They can be combined in a variety of ways with existing permeation systems. ILs will clearly have a large role to play in transdermal drug delivery in the near future. Their use as singular agents remains to be proven; however, when combined with other formulation strategies, the activity enhancing transdermal permeation is remarkably greater.

## Figures and Tables

**Table 1:** Singular change to cation chain length leading to altered physicochemical profile. IL—ionic liquid.

Chain Length Alteration	Change on Physio-Chemical Properties of ILs	Reference
Increase	Increased viscosity	[6,30]
	Increase enthalpy of vaporization	[6]
	Increased aggregation (not necessarily ordered)	[6,30,31]
	Increased toxicity (bacterial and marine ecosystems)	[3,32]
	Increased surfactant activity	[6]
Chain lengths similar to biological membranes	Increased bioaccumulation (potential for toxicity)	[3,20]
Decrease	Increased conductance	[6]
	Increase in electrostatic forces	[6,30]
	Ordered aggregation; depends largely on polarity and geometric packing	[6,31]
	Increased lipase catalytic activity	[19,33]
	Increased polarity	[6]

**Table 2:** Permeation techniques (including formulation enhancement strategies, physical permeation techniques, and chemical permeation enhancers) employed and examples of well-known applications of these techniques.

Permeation Techniques	Example of Technique	References
Formulation enhancement	1) Supersaturated systems 2) Microemulsions 3) Drug moiety modification	[69,70,71]
Physical permeation techniques	1) Electroporation 2) Sonophoresis 3) Microneedles	[72,73,74,75]
Chemical permeation enhancers	1) Alcohols Long-chain fatty alcohols Short-chain alcohols 2) Fatty acids 3) Sulfoxides 4) Terpenes	[57,76,77]

**Table 3:** Presented here are some of the applications of ILs synergistically used to enhance drug delivery with chemical permeation enhancers (CPEs). APIs—active pharmaceutical ingredients.

Chemical Enhancer	ILs	Synergism Documented	Reference
Lipid vesicles	ILs based on methylimidazolium chloride	1) Enhanced permeation 2) Improved therapeutic range 3) Vesicle stabilized	[97]
Surfactants	Dimethyl-imidazolium dimethyl-phosphate	1) Enhanced permeation of sparingly soluble APIs 2) Reduced cytotoxicity	[98]
Terpenes	Menthoxymethyl-3-methylimidazolium chloride	1) Enhanced spatial drug delivery 2) Improved targeting of specified receptors	[99]
Amines	Amine-based ILs	1) Permeation of hydrophobic and hydrophilic molecules 2) No skin tissue injury during permeation	[81]
Alcohols	*N*-*tert*-Alcohol-substituted imidazole	1) Medium for selective reactions 2) Lowered instances of unwanted byproducts	[100]

**Table 4:** The use of IL-APIs with beneficial delivery and therapeutic outcomes.

IL-API Formed	Synergism	Efficacy	Reference
Acetyl salicylic acid/salicylate	Improved manufacturing methods Altered side-effect Profile	Solvent-free synthesis Lowered Gastric distress	[14,109,110,111]
Lidocaine docusate	Improved therapeutic outcome	Longer duration of action	[107,112]
Ranitidine docusate	Improved manufacture outcomes	Improved polymorphic challenges	[107]
Didecyldimethylammonium ibuprofenate	Proof of concept	Dual API formation	[107]
